# Identification of QTL related to anther color and hull color by RAD sequencing in a RIL population of *Setaria italica*

**DOI:** 10.1186/s12864-021-07882-x

**Published:** 2021-07-20

**Authors:** Huifang Xie, Junliang Hou, Nan Fu, Menghan Wei, Yunfei Li, Kang Yu, Hui Song, Shiming Li, Jinrong Liu

**Affiliations:** 1 Anyang Academy of Agriculture Sciences, 455000 Anyang, Henan China; 2 BGI Institute of Applied Agriculture, BGI-Shenzhen, 518120 Shenzhen, Guangdong China

**Keywords:** Foxtail millet (*Setaria italica*), Restriction site-associated DNA sequencing (RADseq), Quantitative trait loci (QTL), Anther color, Hull color, Inconsistent rate analysis, *cinnamyl alcohol dehydrogenase* (*CAD*) gene

## Abstract

**Background:**

Foxtail millet (*Setaria italica*) is one of the oldest domesticated crops and has been considered as an ideal model plant for C_4_ grasses. It has abundant type of anther and hull colors which is not only a most intuitive morphological marker for color selection in seed production, but also has very important biological significance for the study of molecular mechanism of regulating the synthesis and metabolism of flavonoids and lignin. However, only a few genetic studies have been reported for anther color and hull color in foxtail millet.

**Results:**

Quantitative trait loci (QTL) analysis for anther color and hull color was conducted using 400 F_6_ and F_7_ recombinant inbreed lines (RILs) derived from a cross between parents Yugu18 and Jigu19. Using restriction-site associated DNA sequencing, 43,001 single-nucleotide polymorphisms (SNPs) and 3,022 indels were identified between both the parents and the RILs. A total of 1,304 bin markers developed from the SNPs and indels were used to construct a genetic map that spanned 2196 cM of the foxtail millet genome with an average of 1.68 cM/bin. Combined with this genetic map and the phenotypic data observed in two locations for two years, two QTL located on chromosome 6 (Chr6) in a 1.215-Mb interval (33,627,819–34,877,940 bp) for anther color (yellow - white) and three QTL located on Chr1 in a 6.23-Mb interval (1–6,229,734 bp) for hull color (gold-reddish brown) were detected. To narrow the QTL regions identified from the genetic map and QTL analysis, we developed a new method named “inconsistent rate analysis” and efficiently narrowed the QTL regions of anther color into a 60-kb interval (34.13–34.19 Mb) in Chr6, and narrowed the QTL regions of hull color into 70-kb (5.43–5.50 Mb) and 30-kb (5.69–5.72 Mb) intervals in Chr1. Two genes (*Seita.6G228600.v2.2* and *Seita.6G228700.v2.2*) and a *cinnamyl alcohol dehydrogenase* (*CAD*) gene (*Seita.1G057300.v2.2*) with amino acid changes between the parents detected by whole-genome resequencing were identified as candidate genes for anther and hull color, respectively.

**Conclusions:**

This work presents the related QTL and candidate genes of anther and hull color in foxtail millet and developed a new method named inconsistent rate analysis to detect the chromosome fragments linked with the quality trait in RILs. This is the first study of the QTL related to hull color in foxtail millet and clarifying that the *CAD* gene (*Seita.1G057300.v2.2*) is the key gene responsible for this trait. It lays the foundation for further cloning of the functional genes and provides a powerful tool to detect the chromosome fragments linked with quality traits in RILs.

**Supplementary Information:**

The online version contains supplementary material available at 10.1186/s12864-021-07882-x.

## Background

Foxtail millet [*Setaria italica* (L.) P. Beauv.] is one of the oldest domesticated diploid C_4_ crops and has been widely cultivated in northern China for more than 11,500 years [1, 2]. It has characteristics of self-fertilization, relatively small genome size (~ 490 MB), short life cycle (~ 12 weeks), and rich germplasm resources, and has become an ideal model plant for C_4_ grasses [3–7]. Compared with *Arabidopsis thaliana* and *Oryza sativa*, foxtail millet is abiotic stress tolerant particularly to drought and salinity and can make efficient use of light energy. Compared with maize (*Zea mays*), sorghum (*Sorghum bicolor*), and other C_4_ crops, foxtail millet has virtues of higher nutritional value, rich in proteins, folic acid, vitamin E, carotenoids, and selenium [8–10]. In recent years, there has been a great progress in the areas of genomics, functional genomics, and molecular breeding in foxtail millet.

In 2012, a high-quality reference genome sequence of foxtail millet (cultivar Yugu1) was obtained through Sanger sequencing, which covered 80 % of the genome (~ 400 Mb) [11]. At the same time, the draft genome sequence of another cultivar Zhanggu was also completed, with about 423 Mb of the genome assembled and 38,801 genes identified [12]. Until 2020, a reference-grade genome of an indoor-cultivated rapid-cycling mini foxtail millet mutant *Xiaomi* comprising 429.94 Mb of sequences was assembled based on single-molecule real-time subread sequences [3]. Deciphering of the millet genome provides a unique resource for millet genetic breeding. A large number of molecular markers have been developed and utilized, including single-nucleotide polymorphisms (SNPs), indels, structural variants, simple-sequence repeats (SSRs), and Expressed Sequence Tag-SSRs [13–17]. The rapid development of sequencing technology in recent years allowed large-scale whole-genome sequencing becoming possible, and further facilitate numbers of quantitative trait loci (QTL) related to agronomic traits being mapped in *S. italica*, which largely accelerate the molecular breeding process of millet [18–33].

Anther color is one of the important characters of plants. The increase of pigment accumulation is related to the higher photosynthetic rate and the stronger resistance to some biotic and abiotic stress factors [34]. Hull color is also one of the most important characters of grain, which can not only be used as the most intuitive morphological marker for color selection in seed production, but also has a very important biological significance for the molecular mechanism of the genes regulating the synthesis and metabolism of flavonoids and lignin [35]. Anthocyanins are bioactive compounds responsible for the colors of many plant organs such as leaves, flowers, fruits, and roots [36]. Studies in *O. sativa* have shown that the color variation of *O. sativa* is often affected by the composition and metabolism of flavonoids and lignin. Tunen et al. reported that the mutation of chalcone flavanone isomerase resulted in pollen color shifting from yellow to white [37]. Rahim et al. found three *MYB10* genes responsible for anther pigmentation in peach fruit [36]. Cui et al. found the functional alleles of the *Rc* gene conferred proanthocyanidin pigmentation of the pericarp existed in most wild and weedy *Oryzas*, while nonfunctional *rc* alleles were strongly retained during rice domestication [38]. Sun et al. proposed a C-S-A gene model to explain rice hull pigmentation. In this gene system, *C1* encodes a R2R3-MYB transcription factor and acts as a color-producing gene, and *S1* encodes a bHLH protein that functions in a tissue-specific manner; *C1* interacts with *S1* and activates expression of *A1*, which encodes a dihydroflavonol reductase. The functional *A1* leads to purple hull and loss of function of A1 leads to a brown hull color [39]. The mutation of *cinnamyl alcohol dehydrogenase* (*CAD*) genes caused reddish-brown color in leaf midribs and stem sclerenchyma in *Z. mays* [40], brown vascular tissue and altered lignin content in *S. bicolor* [41, 42] and gold hull color in *O. sativa* [35, 43, 44].

The genetic basis of anther color and hull color have rarely been reported in foxtail millet. Ni and Han showed that the genes controlling anther color were located on chromosome 6 (Chr6) of foxtail millet, but the physical positions of the loci identified by these two studies were inconsistent [28, 45]. Ni et al. used whole-genome sequencing technology to re-sequence 184 recombinant inbred lines (RILs) crossed between cultivars Zhanggu and A2 and mapped the QTL related to anther color (yellow - brown) onto the long arm of Chr6 (bin2304: 35,098,775–35,426,784) [28]. Han et al. narrowed the locus related to anther color (yellow - white) onto a genomic region around 94.7 kb at position 34,068,360–34,163,067 in Chr6 by the newly developed indel markers in a F_2_ segregating population (650 lines) derived from crossing of cultivars E1005 and Pinzi 39 [45].

Even with several efforts, the genetic basis of anther and hull color in foxtail millet is still unclear. To dissect the genetic basis of anther color and hull color in foxtail millet, QTL analysis was conducted with 400 RILs developed from a cross between Yugu18 and Jigu19 in two locations for two years to map the QTL related to these two traits. The results of this study will benefit for cloning of functional genes and understanding of the mechanism controlling anther and hull color formation in foxtail millet.

## Results

### Phenotypic evaluation

The parents Yugu18 and Jigu19 had different phenotypes of anther color and hull color. Yugu18 had yellow anthers, and Jigu19 had white anthers. Yugu18 had gold hulls, and Jigu19 had reddish brown hulls (Figure S1). In order to investigate the genetic basic of these two color-related traits, we crossed Yugu18 with Jigu19 and performed self-fertilization of F_1_ progeny for six generations and achieved a population of 400 RILs.

The 400 RILs and their parental lines were planted in two locations (Chengde and Anyang) for two years (2018 and 2019). They exhibited clear phenotypic differences (Table S1). Pearson correlation analysis was performed among these phenotypic traits (Table 1). These two traits were basically stable in different environments, indicating that the RILs were stable and fixed, and that the environment had relatively minor influence on the traits. The traits of anther and hull color were not correlated, indicating that they may be controlled by different genetic loci.

**Table 1:** Correlation coefficients of anther color (AC) and hull color (HC) in different years and locations

Traits	AC19CD	AC19AY	AC18CD	AC18AY	HC19CD	HC19AY	HC18CD	HC18AY
AC19CD	1							
AC19AY	1.00^**^	1						
AC18CD	0.96^**^	0.96^**^	1					
AC18AY	0.97^**^	0.97^**^	0.98^**^	1				
HC19CD	-0.04	-0.04	-0.02	-0.02	1			
HC19AY	-0.04	-0.04	-0.03	-0.03	0.99^**^	1		
HC18CD	-0.02	-0.02	-0.02	-0.02	0.93^**^	0.94^**^	1	
HC18AY	-0.02	-0.02	-0.02	-0.02	0.93^**^	0.94^**^	1.00^**^	1

*CD* Chengde, *AY* Anyang, 18, 2018 (year), 19, 2019 (year)

^**^Significant at *P* < 0.0001

### Sequencing and SNP identification

**Fig. 1:**
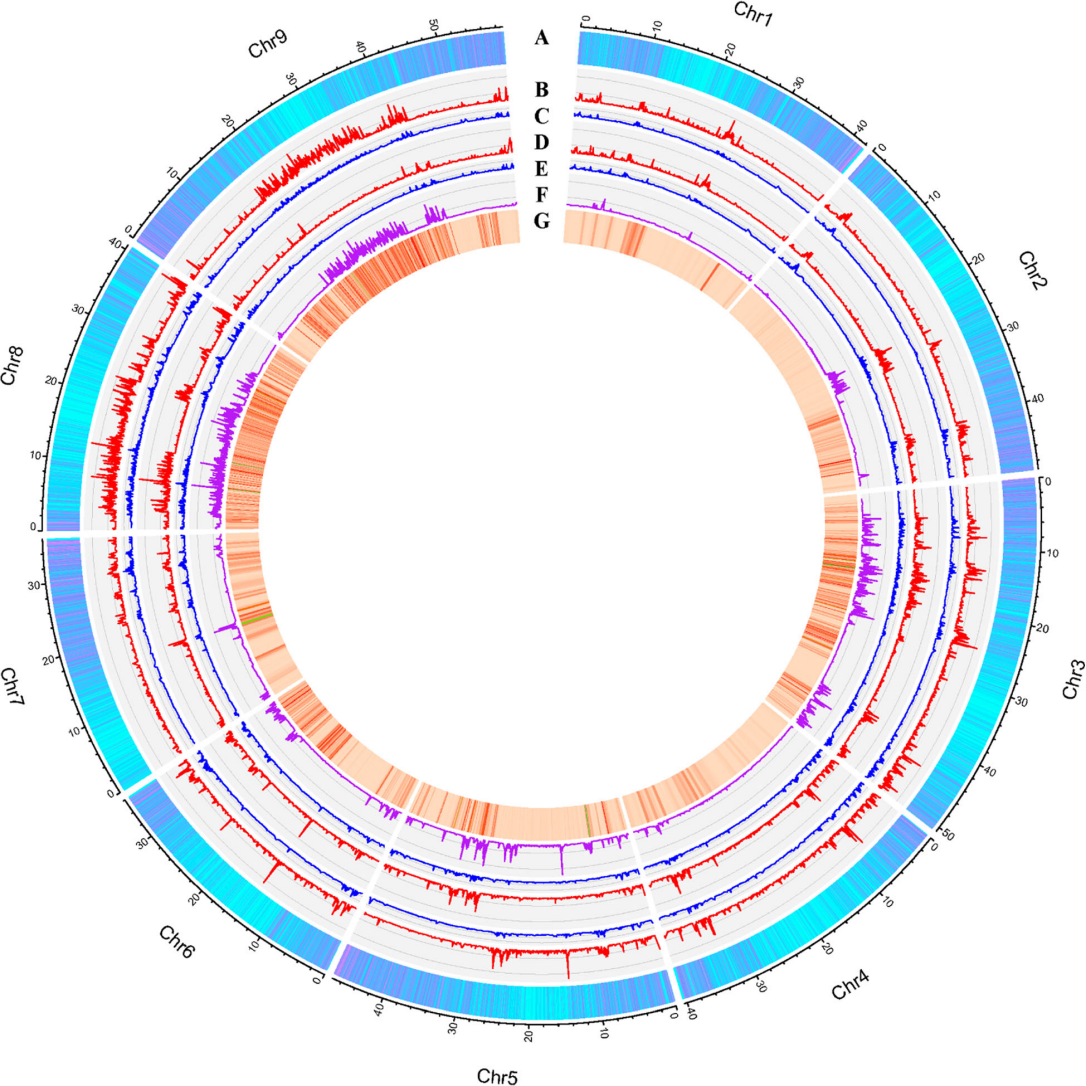
The SNP and indel distribution on chromosomes of the two parents. **A** Gene density in the genome (gene numbers per 50 kb, max = 16); **B** SNPs per 50 kb on Yugu18 (max = 278); **C** indels per 50 kb on Yugu18 (max = 24); **D** SNPs per 50 kb on Jigu19 (max = 399); **E** indels per 50 kb on Jigu19 (max = 28); **F** effective genetic variants between the two parents (max = 154); and **G** genome distributions of the final genetic variations used for bin map construction. Red lines indicate relatively higher variation densities

Restriction-site associated DNA (RAD) sequencing of the parents generated two paired-end libraries with 150-bp reads, including about 1,080 Mb clean data in Yugu18 and 1,377 Mb clean data in Jigu19 (Figure S2). The size of the reference genome of Yugu1 is 403.1 Mb [11]. The sequencing depth of the parental lines was about 2.69-fold in Yugu18 and 3.43-fold in Jigu19 and covered 18.35 and 20.36 % of the whole genome, respectively. The average sequencing depth of each genetic locus which was covered during the alignment was 14.01-fold in Yugu18 and 15.36-fold in Jigu19 (Figure S3). We identified 122,096 SNPs and 9,678 indels between Yugu18 and the reference genome using the SNP identification pipeline, and 112,004 SNPs and 10,857 indels between Jigu19 and the reference genome (Fig. 1). After removing of the genetic variations with no differences between two parents and the genetic loci which were heterozygous or absent in any parent, 74,698 SNPs and 5,737 indels were considered as effective variants (Table S2).

The RAD sequencing of 400 RILs generated 131 Gb of clean next generation sequencing (NGS) data and the average clean data amount was 326.3 Mb (0.8×) with a minimum value of 67.2 Mb and a maximum of 640.3 Mb (Figure S2). About 96.45 % of clean data could be mapped onto the reference genome, which covered 13.89 % of the whole genome and with an average depth of 5.62-fold (Figure S3, Figure S4). The average NGS data set of the parental lines was about four times that of the RILs for higher genome coverage and more accurate genotyping results. The variations were filtered with the parameters of minor allele frequency (MAF) less than 0.1, miss rate less than 50 %, and heterozygosity more than 20 % in the population scale, and 46,023 genetic variations were retained for bin map construction which included 43,001 SNPs and 3,022 indels (Table 2).

**Table 2:** Summary of the bins and genetic variations (GVs) distribution along nine chromosomes of foxtail millet

Chromosome	Length(bp)	Bin number	Linkage(cM)	GVs number	GVs density (/50 kb)
Chr1	42,145,699	76	166.375	467	0.55
Chr2	49,200,776	44	50.75	1402	1.42
Chr3	50,652,576	242	272.681	7983	7.88
Chr4	40,408,058	56	582.516	403	0.50
Chr5	47,253,416	165	246.408	3687	3.90
Chr6	36,015,257	170	272.712	2983	4.14
Chr7	35,964,515	69	116.238	1225	1.70
Chr8	40,690,061	269	170.248	15,315	18.82
Chr9	58,970,518	213	318.173	12,558	10.65
Total	401,300,876	1,304	2,196	46,023	5.73

The variation numbers ranged from 403 for Chr4 to 15,315 for Chr8. The variation density across the chromosomes (variation numbers per 50 kb window) showed an uneven distribution in the whole genome (Fig. 1). While considering the chromosome length differences, the range of the variation density (numbers per 50 kb) on different chromosomes was 0.50–18.82. Chr3, Chr8, and Chr9 possessed more genetic variations (> 7 per 50 kb) than did other chromosomes (< 5 per 50 kb) (Table 2). We calculated the heterozygosity of each sample in the final variations set and discarded 12 samples due to their relatively higher heterozygosity (> 30 %) (Table S3).

### Bin map and genetic map construction

**Fig. 2:**
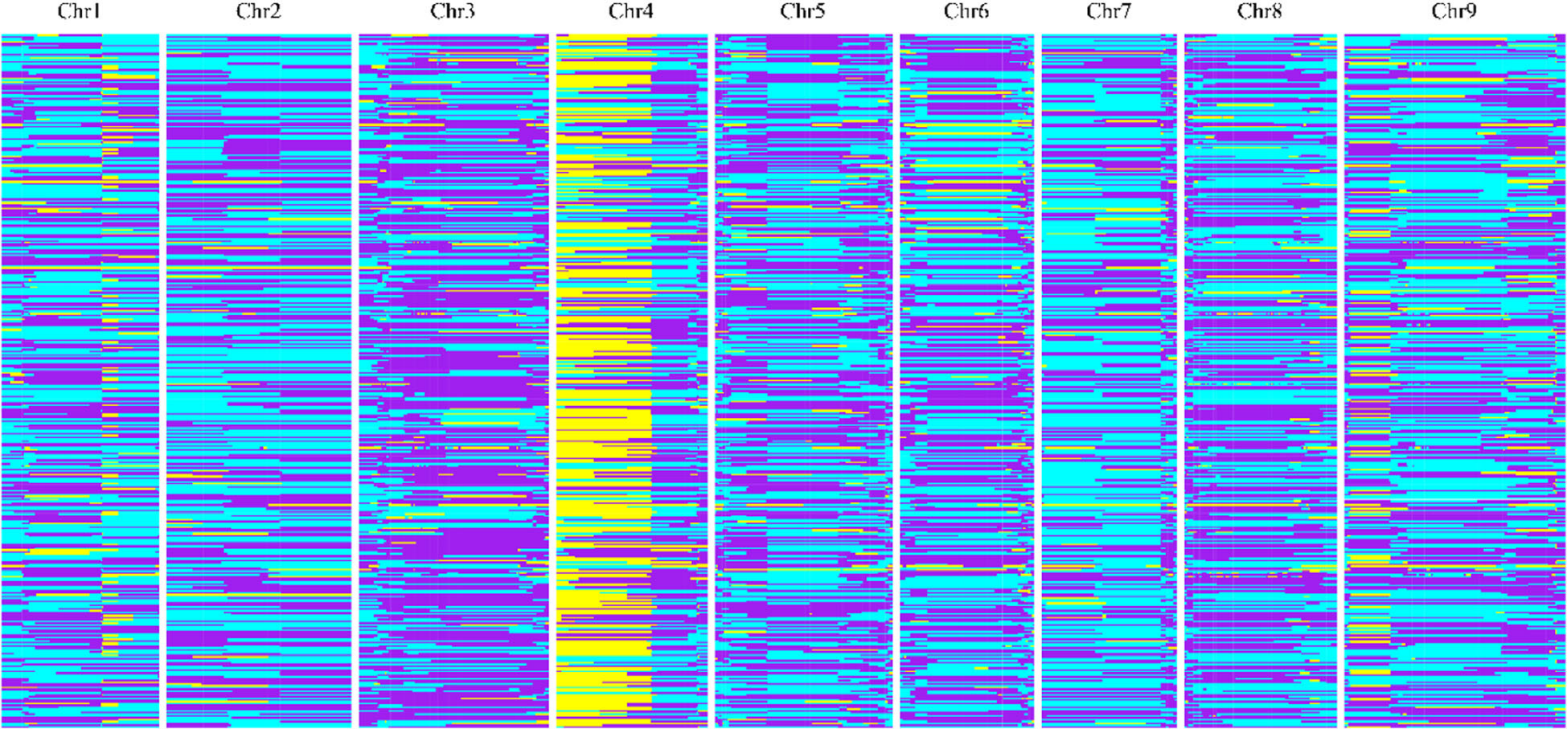
Recombination bin map of 388 foxtail millet RILs. The whole map contained 1,304 bin markers. Purple, genotype of Yugu18; Cyan, genotype of Jigu19; Yellow, heterozygous genotypes. Each row represents the genotype of an individual RIL. Chromosomes are separated by vertical white lines

Bin markers were achieved by sliding 15 SNPs as a window base-by-base to determine the genotype of the window and identify the recombination breakpoints along each chromosome (Fig. 2). We detected 1,304 bin markers in total (Table 2) and constructed a recombination bin map of 388 foxtail millet RILs (Fig. 2). The physical length of the bins ranged from 20.011 kb to 18.557 Mb (Table S4). These bins were regarded as genetic bin makers and were used to construct the linkage map that spanned 2196 cM of the foxtail millet genome with an average bin interval of 1.68 cM. The genetic distances between the adjacent bin markers ranged from 0.10 to 28.69 cM (Table S4). The large fragments of the bins may be due to the lack of genetic diversities between the two parental lines.

**Fig. 3:**
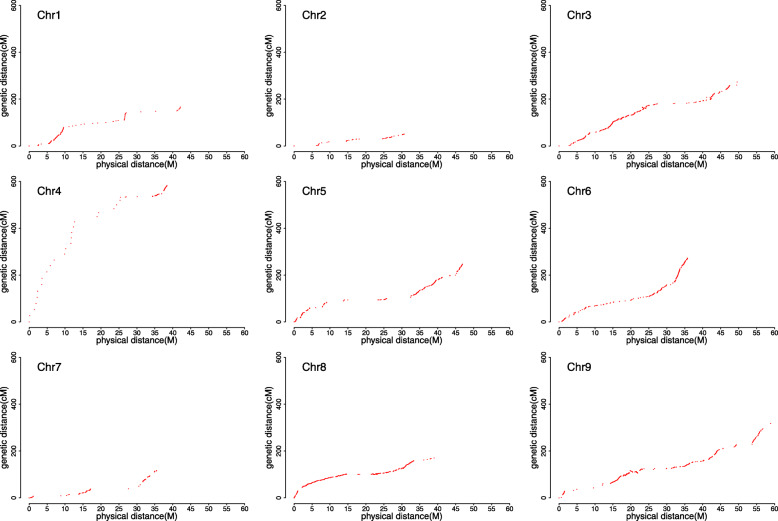
Genetic distance vs. physical distance. Genetic position of the 1,304 bins was plotted against the corresponding physical position. Discontinuous plots show that there were no valid markers in these regions of the chromosome

To evaluate the quality of the bin map build in this study, the collinearity of the bin markers between the genetic positions and their physical locations in the reference genome was conducted by plotting the genetic position of the 1,304 bins against the corresponding physical position (Fig. 3). Our data showed that the genetic and physical positions of the bin markers roughly corresponded, and a large number of discontinuous plots showed that there were no valid markers in these regions of the chromosome. Moreover, short genetic distances were revealed around centromeric regions, where genetic recombination lacked as our expected. Overall, the collinearity analysis indicated that a high-quality of this bin map, although some genomic regions harbored limited bin markers because of genetic diversity between the two parental lines.

### QTL mapping of Anther Color and Hull Color

**Fig. 4:**
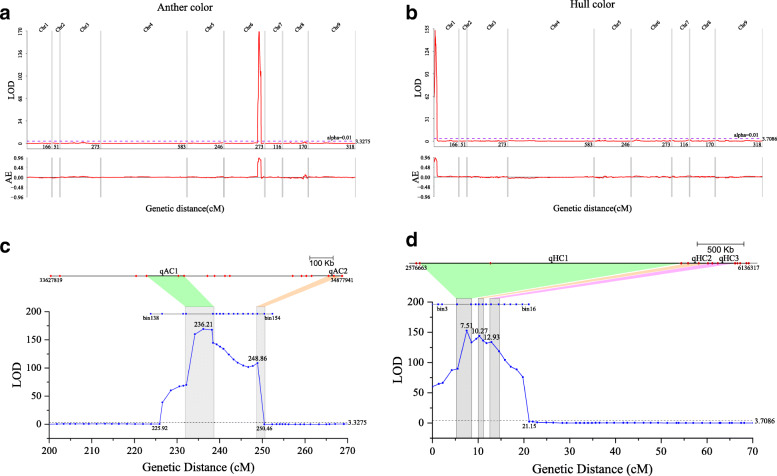
Precise location of the identified QTL region and candidate QTL for the traits of anther color and hull color. **a** QTL region associated with anther color (AC) was located on Chr6 and its additive effect; **b** QTL region associated with hull color (HC) was located on Chr1 and its additive effect; **c** precise location of qAC1 and qAC2 and bin-marker distribution in QTL region on Chr6; **d** precise location of qHC1, qHC2, and qHC3 and bin-marker distribution in QTL region on Chr1. In (**c**) and (**d**), the blue line with blue circular dot represents the LOD distribution, the dark line with blue square dot represents the distribution of bin markers according to the genetic positions, and the dark line with red diamond represents the distribution of bin markers according to the physical positions. The scale of the physical distance is shown in the upper right corner. The recombination bins near the LOD peaks of the QTL are illustrated and linked with colored blocks. Relevant information is also shown in the figure. AE, additive effect.

QTL of anther color and hull color were identified using the composite interval mapping (CIM) method embedded in WinQTLCart2.5 software [46]. With the threshold of LOD > 3 and phenotypic effect (R^2^) > 5 %, only one stable QTL region named qAC spanning 22.28 cM genetic distance, located at the middle-end of Chr6, was detected as the candidate QTL associated with anther color; and one stable QTL region named qHC spanning 21.144 cM genetic distance, located at the beginning of Chr1, was detected as the candidate QTL associated with hull color (Fig. 4). The QTL qAC contained 19 bin markers with 1.215 Mb in the physical interval of 33,627,819–34,877,940 bp on Chr6. The QTL qHC contained 16 bin markers with 6.23 Mb in the physical interval of 1–6,229,734 bp on Chr1. The additive effects of these two QTL regions exceeded 0.95. These results indicate that traits of both anther and hull color were separately controlled by one major gene locus. Additionally, two stable QTL were consistently detected based on the phenotypic data from two field trails for two successive years, indicating that the two traits are primarily genetic controlled and affected little by environmental factors.

According to the bin map and the genetic positions of the LOD peaks in the QTL regions, two QTL (qAC1 and qAC2) were identified in the QTL region qAC and three QTL (qHC1, qHC2 and qHC3) were identified in the QTL region *qHC* using in-house Perl scripts to extract information from the result of WinQTLCart2.5 software (Table 3; Fig. 4). The genetic intervals of the QTL were 0.852–6.254 cM and the physical intervals were 57.926 kb to 2.797 Mb. Interestingly, for the QTL with the longest genetic distance (6.254 cM, qAC1), the physical distance was relatively short (160 kb). We found that the qAC1 locus located the same position as the *Siac1* genetic locus previously mapped using the F_2_ segregating population [45]. Han et al. narrowed this locus into a genomic region around 94.7 kb (Chr6:34,068,360–34,163,067 bp), which was completely covered by the 160.711-kb interval of the qAC1 locus (Chr6:34,039,911–34,200,621 bp). Our finding confirmed the major QTL for anther color in foxtail millet and demonstrated the validity of using this population to identify QTL for other traits.

**Table 3:** QTL for anther color and hull color identified by high-density SNP bin map

Trait	QTL	Chr	Peak(cM)	Marker_interval	Map_interval(cM)	Distance_interval(bp)	LOD	Additive_effect	R^2^(%)
Anther Color	qAC1	6	236.21	Chr6_bin141-Chr6_bin142	232.198-238.452	34,039,911–34,200,621	169.01	0.47	0.89
Anther Color	qAC2	6	248.86	Chr6_bin152-Chr6_bin153	248.847-250.449	34,820,013–34,877,940	108.63	0.43	0.72
Hull Color	qHC1	1	7.51	Chr1_bin4-Chr1_bin5	5.497–8.528	2,615,096–5,411,711	152.64	0.48	0.90
Hull Color	qHC2	1	10.27	Chr1_bin7-Chr1_bin8	10.258–11.110	5,485,413–5,698,001	143.69	0.45	0.82
Hull Color	qHC3	1	12.93	Chr1_bin10-Chr1_bin11	12.918–14.584	5,745,038–5,989,490	133.79	0.45	0.80

*QTL* quantitative trait locus, *Chr* chromosome, *LOD* logarithm of odds difference, *R*^2^ phenotypic variation

### Narrowing QTL regions by the inconsistent rate between the traits and the genotypes in RILs

**Fig. 5:**
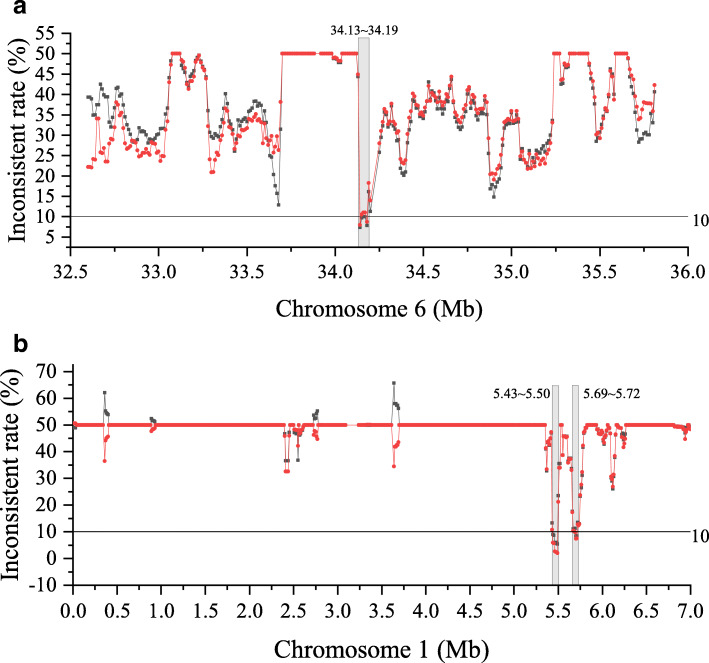
The inconsistent rate between the traits and the genotypes in RILs: **a** anther color and **b** hull color. The average inconsistent rate of certain phenotypic groups was calculated from the SNPs and indels in 50-kb windows with a step of 10 kb. The cutoff of the inconsistent rate was 10 % for both groups. Red line, group of RILs with the Jigu19 phenotype; dark line, group of RILs with the Yugu18 phenotype.

Although we identified QTL regions related to anther and hull color using the constructed genetic map and QTL mapping method, the physical intervals were still too large for further analysis. We developed a new method named inconsistent rate analysis (IRA) to detect the chromosome fragments linked with the quality traits in a RIL population. With this method, we narrowed the QTL regions of anther color into a 60-kb interval (34.13–34.19 Mb) on Chr6, which was completely located in the QTL qAC1 and named it IRA1AC. We also narrowed the QTL regions of hull color into 70-kb (5.43–5.50 Mb) and 30-kb (5.69–5.72 Mb) intervals on Chr1, which overlapped with the QTL qHC2 and were named as IRA2HC and IRA3HC, respectively (Fig. 5).

### Candidate genes for anther color and hull color traits in foxtail millet

There were eight genes located in IRA1AC, twelve genes located in IRA2HC and three genes located in IRA3HC (Table S5). We carefully checked the function of each gene and compared it with the existing research reports. Unfortunately, we did not find any known genes related to anther color in the gene list. This suggested that there may be an unknown gene controlling this trait. However, in reviewing the list of hull color related genes, we found a *CAD* gene(*Seita.1G057300.V2.2*) which was responsible for the changes of the hull colors in rice, should be most likely the key gene accounted for polymorphism of hull color in foxtail millet.

To further identify potential candidate gene in the QTL, we mapped the whole-genome resequencing reads of Yugu18 and Jigu19 to the reference genome and called SNPs and indels using BWA and GATK software. The genes located on the QTL regions and with the SNPs and indels which could cause amino acid changes between the parents were selected as candidate genes for anther color and hull color traits (Table 4). Two genes (*Seita.6G228600.v2.2* and *Seita.6G228700.v2.2*) were identified as candidate genes for the anther color and three genes (*Seita.1G057100.v2.2*, *Seita.1G057300.v2.2*, and *Seita.1G060200.v2.2*) were identified as candidate genes for the hull color. All of these genes had amino acid changes in Jigu19 but no amino acid changes in Yugu18. Most excitingly, we found that the *CAD* (*Seita.1G057300.V2.2*) which was deduced as the key gene for hull color possessed allelic variations in the coding region and amino acid changes between two parental lines.

**Table 4:** Candidate genes for anther color and hull color of foxtail millet

QTL	Gene name	Position (bp)	Reference allele	Variant allele	Variant effect	Description
IRA1AC	*Seita.6G228600.v2.2*	34,176,600	C	T	nonsynonymous	S-acyltransferase 7
	*Seita.6G228700.v2.2*	34,188,715	T	C	nonsynonymous	uncharacterized protein
		34,188,806	G	C	nonsynonymous	
		34,188,814	A	C	nonsynonymous	
		34,188,829	C	CCGCGGAGGG	Non frameshift insertion	
		34,188,867	G	C	nonsynonymous	
		34,188,911	C	A	nonsynonymous	
		34,188,941	T	G	nonsynonymous	
IRA2HC	*Seita.1G057100.v2.2*	5,482,347	G	A	nonsynonymous	UDP-glucose glucosyltransferase
	*Seita.1G057300.v2.2*	5,496,605	A	G	nonsynonymous	cinnamyl alcohol dehydrogenase
IRA3HC	*Seita.1G060200.v2.2*	5,699,160	A	G	nonsynonymous	sucrose-phosphate synthase 2

It could not be ruled out that genetic variations in the promoter region may also affect gene expression and function, thus causing phenotypic changes. Therefore, we provided the raw annovar annotation results of the SNPs and indels in QTL of each parent (Tables S6, S7, S8, S9, S10 and S11) and genetic variation files between two parents in vcf format as supplementary files for further study (Tables S12, S13 and S14).

## Discussion

In this study, phenotypic analysis and QTL mapping were conducted for anther color and grain hull color in foxtail millet, and a candidate causative gene for the hull color, *CAD* which was located at the tip of the short arm of chromosome 1 was identified. A single base at position 268 on the third exon of this gene, *Seita.1G057300.v2.2*, was A in Yugu18 and alternated to G in Jigu19, resulting in the change of its 90th amino acid from isoleucine (I) to valine (V). We predicted the functional structural domain of the protein at the Pfam website (http://pfam.xfam.org/) and found that amino acids 34 to 149 are the Alcohol dehydrogenase GroES-like domain, which is the catalytic core of the enzyme. The mutation of this amino acid may lead to changes in the spatial three-dimensional structure and substrate binding sites of the CAD protein, thus affecting the protein function. According to its functions in *Z. mays*, *S. bicolor* and *O. sativa*, we suggested that the single base variation in *SiCAD* (*Seita.1G057300.v2.2*) is responsible for the golden hull color of Jigu19.

The genomic region of the QTL related to anther color (yellow - white) mapped in this study was close to the locations which were reported in previous studies, but there were still subtle differences. In the previous studies, Ni et al. mapped the QTL related to anther color (yellow - brown) onto the long arm of Chr6 (bin2304: 35,098,775–35,426,784) [28]. Han et al. narrowed the locus related to anther color (yellow - white) onto a genomic region around 94.7 kb at position 34,068,360–34,163,067 in Chr6 [45]. In this study, we mapped this QTL onto a genomic region around 60 kb at position 34,130,000–34,190,000 in Chr6. Although the genomic region we identified does not match with Han et al.’s, there is still a partial overlap. The differences of the genomic regions of the QTL in different studies may be due to differences in mapping populations, errors in phenotypic observations, errors in the sequencing process, and differences in analytical methods.

The ability of using RAD sequencing to directly identify key genes responsible for phenotypic changes of hull color from a RIL isolated population is an ample evidence of the effectiveness of our sequencing strategy and analytical methods. To achieve accurate mapping analysis for the target traits, we took the following approach. First, the accuracy of parental genotypes is crucial for determining the genotypes of a mapping population [47]. For this purpose, we increased the clean data amount of the parental lines. The clean data amount for Yugu18 was 3.3-fold and that of Jigu19 was 4.2-fold compared with the average clean data amount of 326 Mb in RILs, which resulted in 18.35 and 20.36 % coverage of the whole genome, respectively. These were much higher than the average coverage of 13.89 % in the RILs. The average sequencing depth of each genetic locus which was covered during the alignment was 14.01-fold in Yugu18 and 15.36-fold in Jigu19, which was also much higher than the average depth of 5.62-fold in the RILs. Second, we developed a new method, IRA, to detect the chromosome fragments linked with the quality trait in RILs. With this method, we efficiently narrowed the QTL regions of anther color from a 1.215-Mb physical interval into a 60-kb interval on Chr6 and narrowed the QTL regions of hull color from a 6.23-Mb interval into 70-kb and 30-kb intervals on Chr1 (Fig. 5). The assumption was that the genotypes in the phenotype groups should be consistent with the genotypes of the corresponding parents in the target DNA fragments. Ideally, in different phenotypic groups, the chromosome segments that lead to phenotypic differences should be exactly the same as in the parents. Due to the errors of experiment and sequencing, there is always some noise in the actual analysis. However, the smaller the inconsistency, the more likely it is to be the target fragment. We also scanned the whole genome with this method, and only these two genomic regions were identified as candidate QTL (Figures S5 and S6).

Although we hypothesized that the genes corresponding to the loci *Seita.6G228600.v2.2* and *Seita.6G228700.v2.2* are most likely the candidate genes responsible for the anther color, and locus *Seita.1G057300.v2.2* was responsible for the hull color, further gene cloning and functional analysis are planned to reveal the genetic mechanisms accounting for these traits.

## Conclusions

To reveal the genetic basis of anther and hull color traits in foxtail millet, conventional QTL analysis with bin map and genetic map construction, and a newly developed IRA method were employed with 400 F_6_ and F_7_ RILs derived from a cross between parents Yugu18 and Jigu19. The QTL regions identified by these two methods were consistent. However, the interval identified by IRA method was smaller and more accurate, because this method uses more SNP and indel information. We narrowed the QTL regions of anther color into a 60-kb interval (34.13–34.19 Mb) on Chr6 and of hull color into 70-kb (5.43–5.50 Mb) and 30-kb (5.69–5.72 Mb) intervals on Chr1. Two loci of genes (*Seita.6G228600.v2.2* and *Seita.6G228700.v2.2*) and one locus (*Seita.1G057300.v2.2*) with amino acid changes between the parents detected by whole-genome resequencing data were identified as candidate genes for anther and hull color traits, respectively. This is the first study of the QTL related to hull color in foxtail millet and clarifying that the *CAD* gene (*Seita.1G057300.v2.2*) is responsible for this trait. These QTL and genes provide the foundation for further cloning of the functional genes and study of the genetic basis of anther and hull color in foxtail millet.

## Methods

### Plant materials and phenotyping

Yugu18 was selected as the male parent and Jigu19 was selected as the female parent to construct an RIL population by single seed descent strategy. Two sites, Anyang (113°67′E, 35°52′N, Henan Province, China) and Chengde (118°25′E, 40°45′N, Hebei Province, China), were used for planting and phenotypic identification and the F_6_ and F_7_ generation populations were grown separately in 2018 and 2019. The plants grown in Anyang in 2019 were used for DNA extraction and genome sequencing. The color traits of anther and hull were characterized at the flower stage and when millet was ripe and harvested, respectively. Correlations were analyzed by Pearson’s correlation between different years and locations of the same trait [48].

### Sequencing of parental lines and RIL population

Total genomic DNA was extracted from young leaf tissues of the parental lines and F_7_ population with the CTAB method [49]. DNA was quality-controlled and quantified using 1.3 % agarose gel electrophoresis and NanoDrop™ One UV-Vis spectrophotometer (Thermo Fisher Scientific, 

USA). The DNA concentration should be greater than 50 ng/µL and the total DNA amount should be greater than 1µg. RADseq libraries were constructed according to a previous protocol [50, 51]. Briefly, the genomic DNA was digested using restriction enzyme *Taq*I (5’-TCGA-3’, New England Biolabs) at 65 ℃ for 20 min and ligated with P1 adapters. Every 24 samples of DNA were pooled together and were purified and recovered using QIA quick Gel Extraction Kit. DNA fragments of 350 to 550 bp were isolated using a high throughput DNA fragment recovery system (Pippin HT, Sage Science, USA) and quantified using a Qubit® 3.0 fluorimeter (Invitrogen Ltd, Paisley, UK), respectively. DNA amount should be greater than 15ng and the volume should be less than 23 µL. A divergent adapter P2 was ligated to the obtained DNA fragments. Samples were purified again, and 20 ng of this product was used in a PCR amplification with 20 µL Phusion Master Mix, 5 µL of 10 µM modified amplification primer mix, and up to 50 µL with H_2_O. PCA products from each library were purified by magnetic beads and quantified using a Qubit fluorimeter (DNA concentration should be 0.3 ng/µL, the length of DNA fragment should be 300 to 500 bp and no dimer primers or other contaminants). The RADseq libraries were sequenced for paired-end (PE) 100-bp reads in BGI-Shenzhen (Shenzhen, Guangdong, China) on BGISEQ 500 instrument. Low-quality reads, reads with adaptor sequences, and duplicated reads were filtered using SOAPnuke [52], and the remaining high-quality data were used for further bioinformatics analysis.

### Sequence alignment, genotyping, and recombination breakpoint determination

Reads of all samples were mapped to the reference genome sequence of *S. italica* (Setaria_italica_v2.0) using BWA software (Ver. 0.7.17) [53]. The mapping rate and coverage were calculated by Samtools [54] and ReSeqTools [55]. The SNPs and indels were then identified from alignment by GATK tools (V4.0) [56] and filtered with the following parameters: QD < 2.0, MQ < 40.0, MQRankSum < − 12.5, and ReadPosRankSum < − 8.0. Then the common genetic variations between the parents and the genetic loci that were heterozygous or absent in any parent lines were removed. At the population scale, the variations with an MAF less than 0.1, miss rate less than 50 %, and heterozygosity more than 20 % were discarded [57]. Bin markers were achieved by sliding 15 SNPs as a window base-by-base to determine the genotype of the window and identify the recombination breakpoints on the chromosomes [28, 58, 59]. The genotype of the window was identified according to consistency with that of the parent and with 70 % as the cutoff. If more than 70 % of the variants in a window were consistent with Yugu18, the window was called Yugu18 genotype. If more than 70 % of the variants in a window were consistent with Jugu19, the window was called Jigu19 genotype. Otherwise, the genotype was called heterozygous genotype [28]. The breakpoint was resolved at the boundary of Yugu18, Jigu19, and heterozygous genotypes.

### Genetic map construction and QTL mapping

The phenotype of each RIL and genotype of each bin were collected for gene mapping and QTL analysis. MSTmap [60, 61] was used to construct the linkage map and recombination frequencies were converted into cM using the Kosambi algorithm. WinQTLCart (V2.5_011) software [46] was used to detect QTL using the CIM method. Each group of phenotypic data was iterated 1,000 times to calculate the *P*-value, and QTL were called for LOD > 3.0.

### Inconsistent rate analysis

We divided RILs into two groups (A and B) based on phenotypes derived from the parents. Only those loci that were homozygous in parents and less than 50 % in both deletion and heterozygosity in the population could be used for IRA analysis. The inconsistent rate of group A in a SNP locus was defined as sample numbers with genotype B/(sample numbers with genotype A and genotype B) in the RILs with phenotype (A) The inconsistent rate of group B in an SNP locus was defined as sample numbers with genotype A/(sample numbers with genotype A and genotype B) in the RILs with phenotype (B) We calculated the average inconsistent rate of different phenotypic groups in a 50-kb window with a step of 10 kb sliding along the chromosome.

### Identification of candidate genes in the QTL

The genes in the QTL were extracted from the genome annotation version Sitalica_312_v2.2 of Phytozome v13 [62]. The functions of genes were annotated by mapping the genes to the Nr databases using the software BLAST (v2.2.26). The whole-genome resequencing short reads were downloaded from NCBI with the accession of Run:SRR13414425 as Jigu19 and Run:SRR13414474 as Yugu18 under the project accession of SRA:SRP301361 [33]. The SNPs and indels were called using BWA and GATK pipeline. The functions of genetic variations located in the QTL regions were annotated by ANNOVAR software [63].

The experiments in this study did not involve endangered or protected species. No specific permits were required for these locations/activities. We declare that all the materials and methods in this study complied with relevant institutional, national, and international guidelines and legislation.

## Supplementary Information

**Additional file 1: Table S1.** Anther and hull color in Yugu18, Jigu19 and RILs. **Table S2.** Number of SNPs, indels and effective SNPs on nine chromosomes in two parents by aligning against reference genome. **Table S3.** Heterozygosity of each sample in the final variations set. **Table S4.** Detailed bin information. **Table S5.** The gene list and functional annotation of QTL related to anther color and hull color in millet. **Table S6.** The annovar annotation result of the SNPs and indels in QTL IRA1AC of Jigu19. **Table S7.** The annovar annotation result of the SNPs and indels in QTL IRA1AC of Yugu18. **Table S8.** The annovar annotation result of the SNPs and indels in QTL IRA2HC of Jigu19. **Table S9.** The annovar annotation result of the SNPs and indels in QTL IRA2HC of Yugu18. **Table S10.** The annovar annotation result of the SNPs and indels in QTL IRA3HC of Jigu19. **Table S11.** the annovar annotation result of the SNPs and indels in QTL IRA3HC of Yugu18. **Table S12.** The SNPs and indels located in the QTL of IRA1AC in vcf format. **Table S13.** The SNPs and indels located in the QTL of IRA2HC in vcf format. **Table S14.** The SNPs and indels located in the QTL of IRA3HC in vcf format.

**Additional file 2: Figure S1.** Anther color and hull color in Yugu18 and Jigu19. **Figure S2.** Sequencing clean data of the parents and the RILs. **Figure S3.** Alignment statistics of the parents and the RILs. **Figure S4.** Distribution of the depth information while the short reads mapped to the reference genome in the parental lines and RILs. **Figure S5.** Identification of QTL related to anther color in RILs with Inconsistent Rate Analysis (IRA) method. **Figure S6.** Identification of QTL related to hull color in RILs with Inconsistent Rate Analysis (IRA) method.

## Data Availability

The dataset supporting the conclusions of this article has been deposited into CNGB Sequence Archive (CNSA) of China National GeneBank DataBase (CNGBdb) [64] with accession number CNP0001799 (https://db.cngb.org/search/project/CNP0001799/).

## Abbreviations

QTL: Quantitative trait locus
RAD: Restriction-site associated DNA
RIL: Recombinant inbreed line
SNP: Single-nucleotide polymorphism
Chr: Chromosome
SSR: Simple-sequence repeat
MAF: Minor allele frequency
CIM: Composite interval mapping
IRA: Inconsistent rate analysis
CAD: Cinnamyl alcohol dehydrogenase
