# Supplementary material for: Identification of QTL related to anther color and hull color by RAD sequencing in a RIL population of Setaria italica
Source: BMC Genomics. 2021 Jul 20;22:556. doi: 10.1186/s12864-021-07882-x (PMC8290542; doi:10.1186/s12864-021-07882-x)
Supplement: Supplementary file 2 — Additional file 2: Figure S1. Anther color and hull color in Yugu18 and Jigu19. Figure S2. Sequencing clean data of the parents and the RILs. Figure S3. Alignment statistics of the parents and the RILs. Figure S4. Distribution of the depth information while the short reads mapped to the reference genome in the parental lines and RILs. Figure S5. Identification of QTL related to anther color in RILs with Inconsistent Rate Analysis (IRA) method. Figure S6. Identification of QTL related to hull color in RILs with Inconsistent Rate Analysis (IRA) method. [file 12864_2021_7882_MOESM2_ESM.docx]

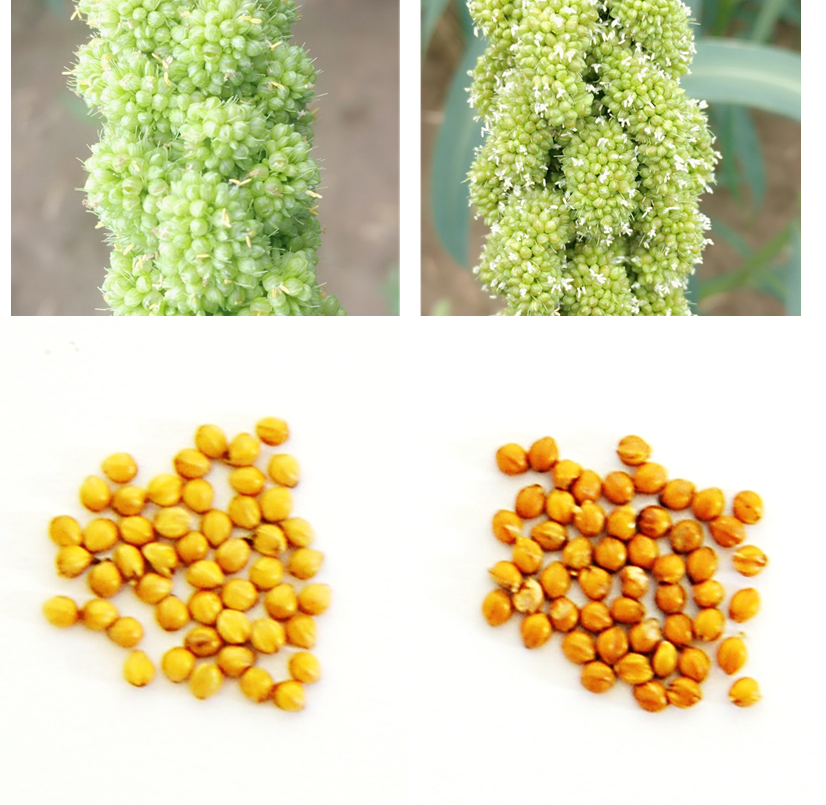


Figure S1. Anther color and hull color in Yugu18 and Jigu19. Left was Yugu18 and Right was Jigu19. Yugu18 had yellow anthers, and Jigu19 had white anthers. Yugu18 had gold hulls, and Jigu19 had reddish brown hulls.


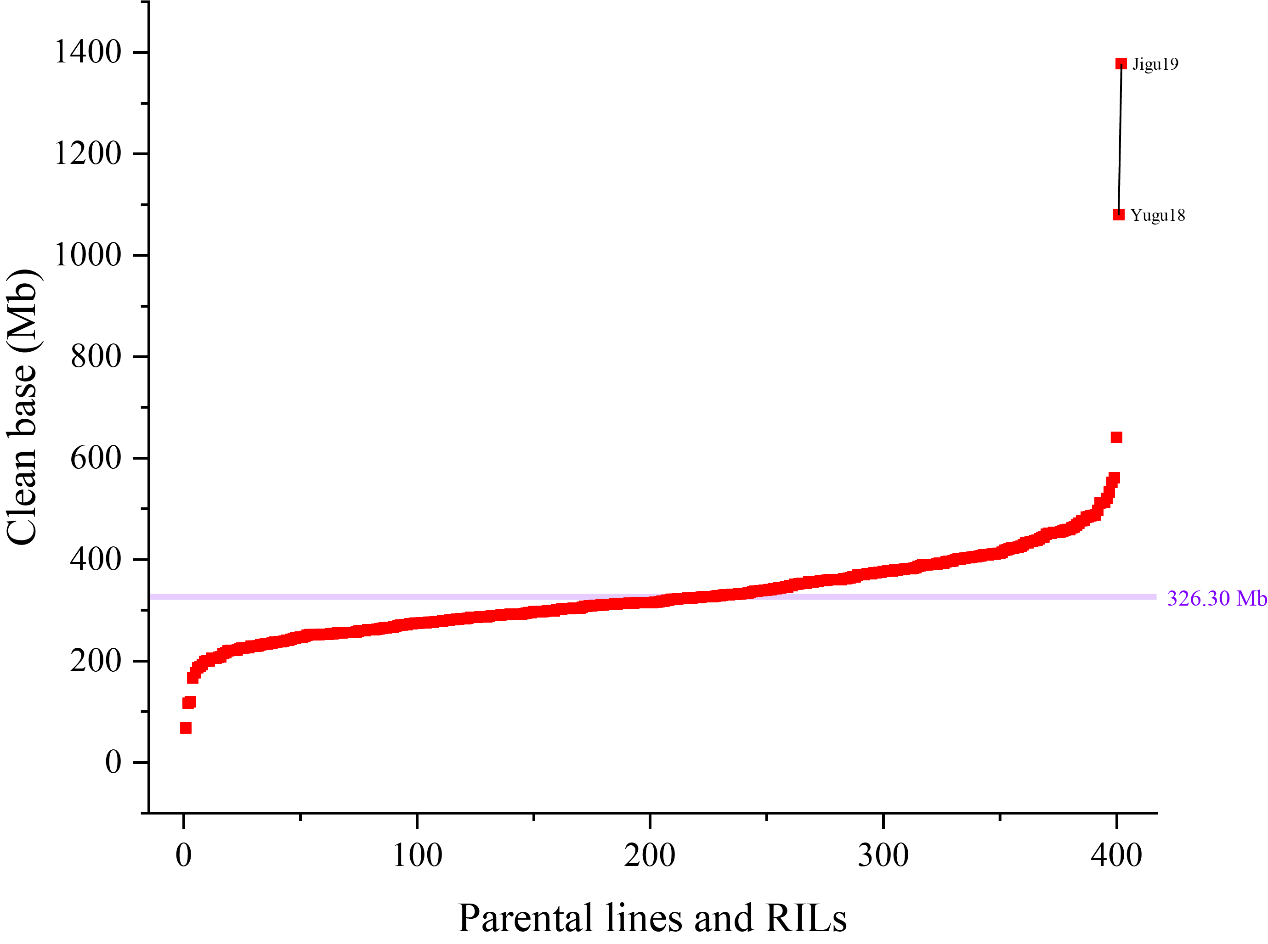


Figure S2. Sequencing clean data of the parents and the RILs. The average data amount was 326.30 Mb in the RIL populations, which was about 0.8x of the whole genome. The parents (Jigu19 and Yugu18) were with obviously higher data amount.


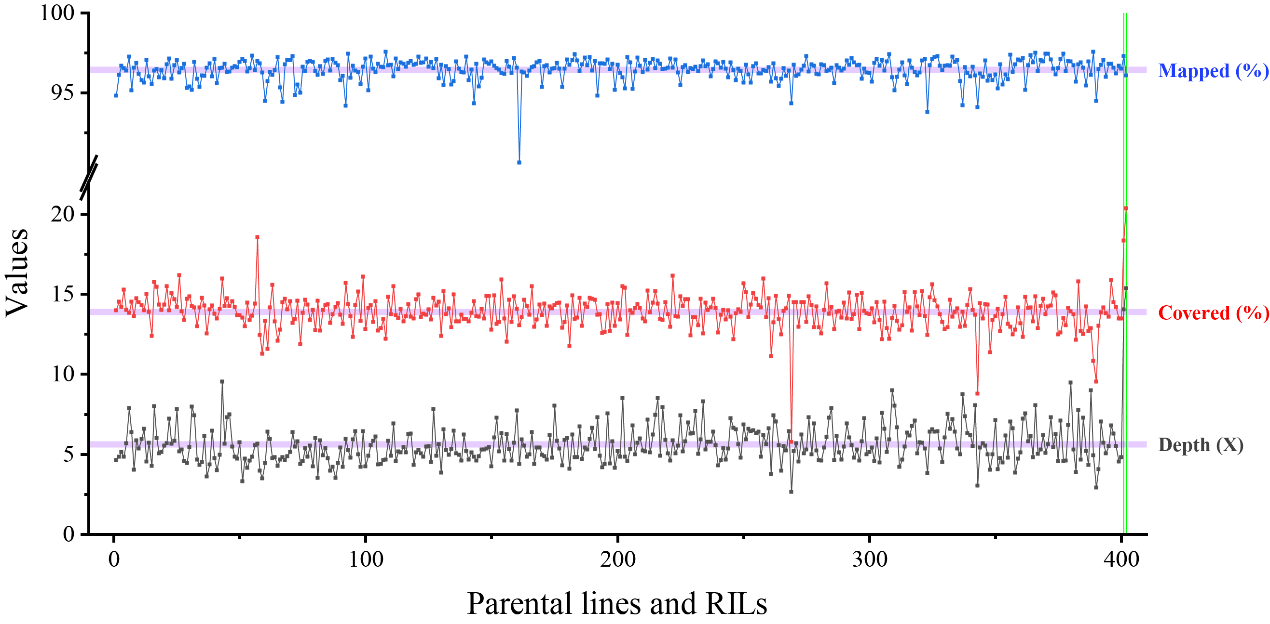


Figure S3. Alignment statistics of the parents and the RILs. From the top to the bottom, the blue line represented the mapping ratio of the clean reads which could be mapped to the genome and the lavender line represented an average value of 96.45%; the red line represented the proportion of the genome which could be covered by the clean reads during the alignment, the lavender line represented an average value of 13.89; the dark line represented the average sequencing depth of each genetic locus which was being covered during the alignment, the lavender line represented an average value of 5.62. The green vertical lines indicated that the parents were with obviously higher sequencing depth and genome coverage.


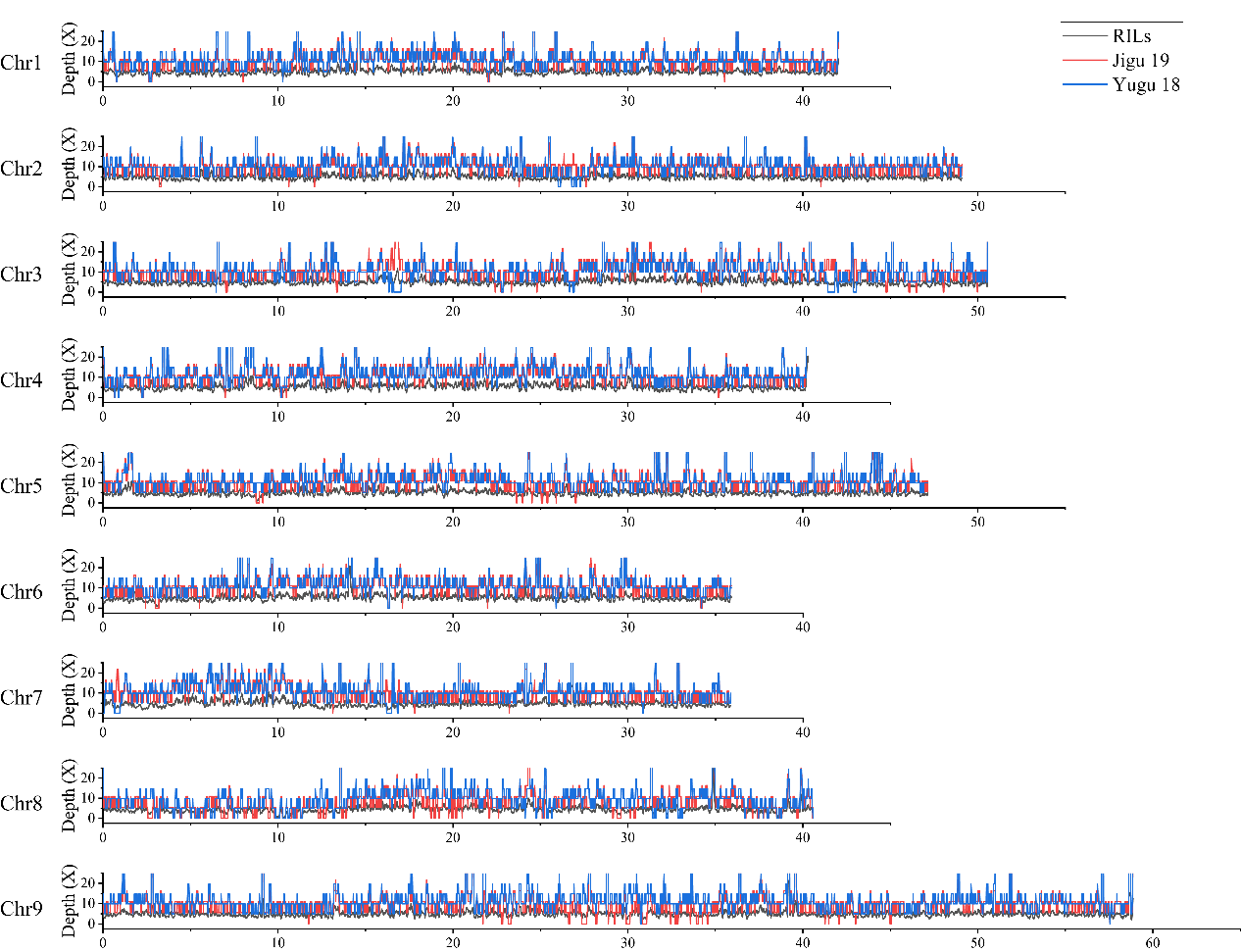


Figure S4. Distribution of the depth information while the short reads mapped to the reference genome in the parental lines and RILs. The ordinate is the depth information. The average depth of RILs was about 5 X and that of the parent lines was about 10 X. The distribution was generally uniform.

Figure S5. Identification of QTLs related to anther color trait in RILs with Inconsistent Rate Analysis (IRA) method. The window was 50 Kb and the step was 10 Kb. The cut-off was 10%. Only the genomic regions with the Inconsistent Rate of both groups under the cut-off were considered as candidate QTLs. Blue arrow indicated there was only one DNA region identified.

Figure S6. Identification of QTLs related to hull color trait in RILs with Inconsistent Rate Analysis (IRA) method. The window was 50 Kb and the step was 10 Kb. The cut-off was 10%. Only the genomic regions with the Inconsistent Rate of both groups under the cut-off were considered as candidate QTLs. Blue arrow indicated there was only one DNA region identified.
